# *AKNA* Frameshift Variant in Three Dogs with Recurrent Inflammatory Pulmonary Disease

**DOI:** 10.3390/genes10080567

**Published:** 2019-07-26

**Authors:** Petra Hug, Linda Anderegg, Alexandra Kehl, Vidhya Jagannathan, Tosso Leeb

**Affiliations:** 1 Institute of Genetics, Vetsuisse Faculty, University of Bern, 3001 Bern, Switzerland; 2 Laboklin, 97688 Bad Kissingen, Germany

**Keywords:** *Canis lupus familiaris*, dog, whole genome sequence, animal model, AT-hook transcription factor, immunology, inflammation, infection, rare disease, precision medicine

## Abstract

We investigated three related Rough Collies with recurrent inflammatory pulmonary disease. The clinical symptoms were similar to primary ciliary dyskinesia (PCD). However, the affected dogs did not carry any known pathogenic PCD variants. Pedigree analysis suggested a recessive mode of inheritance. Combined linkage and homozygosity mapping in three cases and seven non-affected family members delineated 19 critical intervals on 10 chromosomes comprising a total of 99 Mb. The genome of one affected dog was sequenced and compared to 601 control genomes. We detected only a single private homozygous protein-changing variant in the critical intervals. The detected variant was a 4 bp deletion, c.2717_2720delACAG, in the *AKNA* gene encoding the AT-hook transcription factor. It causes a frame-shift introducing a premature stop codon and truncates 37% of the open reading frame, p.(Asp906Alafs*173). We genotyped 88 Rough Collies consisting of family members and unrelated individuals. All three available cases were homozygous for the mutant allele and all 85 non-affected dogs were either homozygous wildtype (*n* = 67) or heterozygous (*n* = 18). AKNA modulates inflammatory immune responses. *Akna^−/−^* knockout mice die shortly after birth due to systemic autoimmune inflammatory processes including lung inflammation that is accompanied by enhanced leukocyte infiltration and alveolar destruction. The perfect genotype-phenotype association and the comparative functional data strongly suggest that the detected *AKNA*:c.2717_2720delACAG variant caused the observed severe airway inflammation in the investigated dogs. Our findings enable genetic testing, which can be used to avoid the unintentional breeding of affected puppies.

## 1. Introduction

Inherited forms of recurrent pneumonia or inflammatory airway disease are often due to primary ciliary dyskinesia (PCD) in humans and domestic animals. PCD is characterized by a loss of function of the motile cilia, not only in the lung, but also in the paranasal sinuses, the middle ear, sperm, the female reproductive tract and the ependyma of the brain. A defect in the structure or the function of the cilia typically leads to recurring infections of the upper and lower respiratory tract. The underlying pathogenesis is a decreased mucociliary clearance of dust and infectious agents [1]. Variants in over 40 genes are known to cause PCD in humans [2,3,4]. PCD causative genetic variants were also reported in purebred dogs. Variants in *CCDC39* [5] and *NME5* [6] were identified in PCD affected Old English Sheepdogs and Alaskan Malamutes, respectively. Despite the extensive knowledge about PCD, there are also clinically described forms of inherited recurrent inflammatory airway disease in humans and dogs, whose underlying genetic cause is still unknown [7,8]. Changes to the cilia may also result from respiratory disease and are then termed secondary ciliary dyskinesia (SCD). Discrimination of PCD and SCD based on clinical signs and/or pathological findings can be challenging and is not routinely available in veterinary medicine [9].

Dog owners recently noticed several closely related Rough Collies with recurrent pulmonary disease, which clinically resembled canine forms of PCD. The goal of this study was to identify the underlying causative genetic defect.

## 2. Materials and Methods

### 2.1. Ethics Statement

All animal experiments were performed according to local regulations. All dogs in this study were privately owned and examined with the consent of their owners. The “Cantonal Committee for Animal Experiments” approved the collection of blood samples (Canton of Bern; permit 75/16).

### 2.2. Animal Selection

This study included 88 Rough Collies. Three related Rough Collies, two of them being half siblings, had recurrent pulmonary disease as reported by the owners. The other 85 dogs were reported as unaffected by their owners. EDTA blood samples were collected for DNA isolation. We also used 539 dogs of various other breeds, which had been donated to the Vetsuisse Biobank (Table S1). They represented population controls without reports of recurrent pulmonary diseases.

### 2.3. DNA Extraction and Genotyping

Genomic DNA was isolated from EDTA blood with the Maxwell RSC Whole Blood Kit using a Maxwell RSC instrument (Promega, Dübendorf, Switzerland). Ten Rough Collies consisting of 3 cases and 7 control dogs from an extended family were genotyped on the Illumina canine_HD BeadChips containing 220,853 markers (Neogen, Lincoln, NE, USA).

### 2.4. Linkage and Homozygosity Mapping

The genotype data of 3 non-affected parents, 2 affected and 4 non-affected offspring in two litters were used for a parametric linkage analysis. Using PLINK v 1.09 [10], markers that were non-informative, located on the sex chromosomes, or missing in any of the nine dogs, containing Mendel errors, or having a minor allele frequency <0.05 were excluded. The final dataset contained 65,446 markers. Parametric linkage analysis using an autosomal recessive inheritance model with full penetrance and a disease allele frequency of 0.4 was performed with the Merlin software [11].

For homozygosity mapping, the genotype data of the three affected dogs were used. The --homozyg and --homozyg -group options in PLINK were used to search for extended regions of homozygosity >1 Mb. The output intervals were matched against the intervals from linkage analysis in Excel spreadsheets to find overlapping regions. The raw single nucleotide variant (SNV) genotypes are available in S1. The output of the linkage and homozygosity mapping is given in Table S2.

### 2.5. Whole Genome Sequencing of an Affected Rough Collie

An Illumina TruSeq PCR-free DNA library with 500 bp insert size of an affected Rough Collie (CL013) was prepared. We collected 227 million 2 × 150 bp paired-end reads on a NovaSeq 6000 instrument (26.9× coverage). Mapping and alignment were performed as described [12]. The sequence data were deposited under study accession PRJEB16012 and sample accession SAMEA4867926 at the European Nucleotide Archive.

### 2.6. Variant Calling

Variant calling was performed using GATK HaplotypeCaller [13] in gVCF mode as described [9]. To predict the functional effects of the called variants, SnpEff [14] software together with NCBI annotation release 105 for the CanFam 3.1 genome reference assembly was used. For variant filtering we used 601 control genomes, which were either publicly available [15] or produced during other projects of our group [16] (Table S3).

### 2.7. Gene Analysis

We used the dog CanFam 3.1 reference genome assembly for all analyses. Numbering within the canine *AKNA* gene corresponds to the NCBI RefSeq accessions XM_014117950.2 (mRNA) and XP_013973425.1 (protein).

### 2.8. Sanger Sequencing

The *AKNA*:c.2717_2720delACAG variant was genotyped by direct Sanger sequencing of PCR amplicons. A 305 bp (or 301 bp for the deletion allele) PCR product was amplified from genomic DNA using AmpliTaqGold360Mastermix (Thermo Fisher Scientific, Waltham, MA, USA) together with primers 5′-CCT GTG AGC CTC TGG AAT GT-3′ (Primer F) and 5′-CTG AGA ATG CCC AGA CCA TC-3′ (Primer R). After treatment with exonuclease I and alkaline phosphatase, amplicons were sequenced on an ABI 3730 DNA Analyzer (Thermo Fisher Scientific). Sanger sequences were analyzed using the Sequencher 5.1 software (GeneCodes, Ann Arbor, MI, USA).

## 3. Results

### 3.1. Phenotype Description

The owners reported recurrent foamy vomiting, shallow breathing, coughing, increased breathing sounds, nasal discharge and fever in three related Rough Collies. Bronchopneumonia was diagnosed by a private veterinarian. The clinical signs started at a few days of age in two affected dogs. The dogs responded to therapy with antibiotics and secretolytics. However, they tended to relapse quickly. Two dogs were reported to be alive at three years with frequent yellowish nasal discharge. No information on the course of the disease is available for the third affected dog.

### 3.2. Genetic Analysis

**Figure 1:**
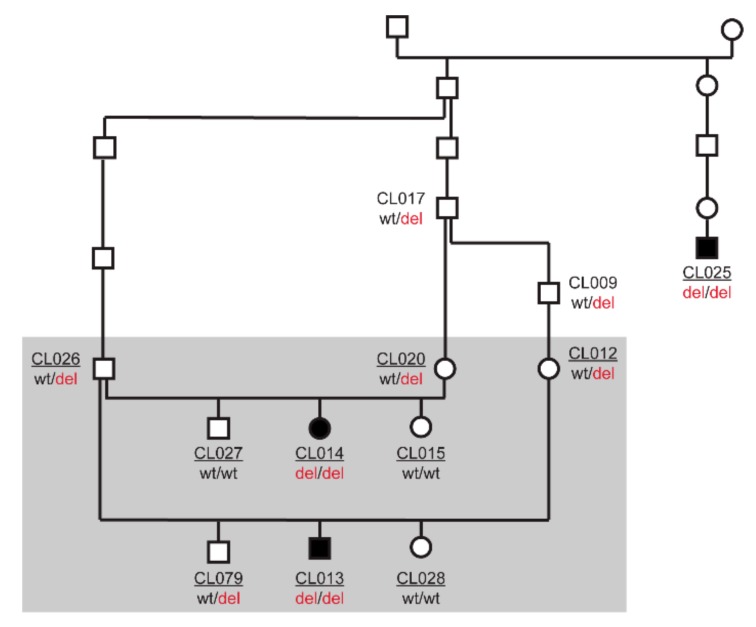
Pedigree of the three investigated cases. Filled symbols represent dogs with recurrent inflammatory pulmonary disease. Open symbols represent non-affected dogs. *AKNA*:c.2717_2720delACAG genotypes are indicated for all dogs, from which a DNA sample was available. The laboratory identifiers of the 10 dogs that were genotyped on the SNV microarray for linkage and homozygosity mapping are underlined. The grey rectangle indicates the 9 dogs that were used for linkage analysis.

Three related cases were reported. The pedigree of these dogs is shown in Figure 1 and suggested autosomal recessive inheritance. Linkage analysis using two litters with six offspring (two affected, four non-affected) and their three parents showed 18 linked genome segments totaling 246 Mb. We additionally performed homozygosity mapping in the three affected dogs. They shared 87 homozygous segments with identical alleles totaling 354 Mb. Intersecting the linked and homozygous regions delineated critical intervals comprising 19 segments on 10 chromosomes spanning 99 Mb or roughly 4.1% of the 2.4 Gb dog genome. The two known canine PCD causative variants, *CCDC39*:c.286C>T [5] and *NME5*:c.43delA [6] were not located in the critical intervals and could thus be excluded.

We sequenced the genome of an affected dog at 26.9× coverage and called SNVs and short indels with respect to the CanFam3.1 genome assembly. We then searched for private homozygous variants in the genome sequence of the affected dog that were not present in 601 control dogs of different breeds (Table 1). We identified only one private homozygous protein-changing variant in the critical intervals (Table S4).

**Figure 2:**
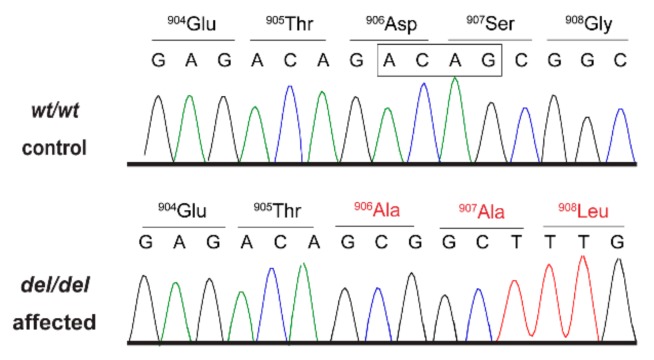
Details of the *AKNA*:c.2717_2720delACAG variant. Representative electropherograms of a control and an affected dog are shown. The four deleted bases are indicated by the square. Altered amino acid translations are shown in red.

This variant represented a 4 bp deletion that can be designated as Chr11:68,576,241_68,576,244delCTGT (CanFam 3.1 assembly). It was located in the *AKNA* gene encoding AT-hook transcription factor and is predicted to result in a frameshift of the coding sequence, XM_014117950.2:c.2717_2720delACAG. The variant introduces a premature stop codon. Due to a lack of suitable RNA samples we did not experimentally investigate whether the mutant transcripts are subject to nonsense-mediated decay and/or nonsense-mediated alternative splicing. If expressed, the predicted translation product of the mutant transcript would lack 529 amino acids of the 1434 amino acid wildtype AKNA protein and contain 172 new amino acids at its C-terminus. The formal variant designation is XP_013973425.1:p.(Asp906Alafs*173). We confirmed the presence of the *AKNA*:c.2717_2720delACAG variant in genomic DNA samples by Sanger sequencing (Figure 2).

We genotyped a total of 627 dogs for the variant and observed perfect genotype-phenotype concordance (Table 2). All three affected Rough Collies carried the deletion in the homozygous state. Eighteen non-affected dogs were heterozygous and presumed carriers. The remaining 67 non-affected Rough Collies were homozygous wildtype. As we tested only a relatively small number of Rough Collies including many close relatives of the affected dogs, we cannot reliably estimate the allele frequency in the breed. The mutant allele at the *AKNA*:c.2717_2720delACAG variant was absent from 539 additionally genotyped dogs of various breeds (Table S1).

## 4. Discussion

In this study, we identified *AKNA*:c.2717_2720delACAG as a candidate causative variant for an inherited form of recurrent inflammatory pulmonary disease in dogs. The affected dogs were initially submitted with a suspected diagnosis of PCD. We therefore were surprised that the candidate causative variant turned out to be unrelated to cilial function. The clinical phenotype has not yet been characterized in detail. We putatively propose the term recurrent inflammatory pulmonary disease to emphasize the phenotypic similarities of the affected dogs with *Akna^−/−^* knockout mice (see below).

*AKNA* encodes the AT-hook transcription factor, which has an important regulatory function in the immune system. It modulates the transcription of CD40 and its ligand CD154 (also called CD40L) in immune cells [17]. The interaction of CD40 and CD154 has been shown to be involved in the negative regulation of T cell autoreactivity and abnormalities in their interaction may lead to autoimmunity. In human systemic autoimmune diseases such as Vogt-Koyanagi-Harada syndrome, AKNA was found to be downregulated in CD4^+^ T-lymphocytes [18]. Furthermore, associations between *AKNA* gene variants with knee osteoarthritis [19] and an elevated risk for cervical cancer due to a deregulation in the inflammasome network were reported [20,21]. To the best of our knowledge, no human patients affected by recurrent inflammatory pulmonary disease or any other pathology with germline coding variants in *AKNA* have been described in the scientific literature. Data from more than 120,000 humans in the gnomAD browser [22] indicate that *AKNA* loss of function (LoF) variants are less frequent than expected (19 observed vs 61.4 theoretically expected LoF variants). No individuals carrying an *AKNA* LoF variant in homozygous state have been identified so far.

*Akna^−/−^* knockout mice are smaller than their wildtype littermates and they die within 10 days after birth due to systemic inflammation, predominantly seen in the lungs [23]. Enhanced leukocyte infiltration and a massive overproduction of pro-inflammatory cytokines and matrix metallproteinases lead to alveolar destruction in the *Akna^−/−^* knockout mice. The murine *Akna* gene contains 22 exons and two different knockout strains were investigated. The initial strain was constructed by deleting exons 19–21. It was not assessed whether a C-terminally truncated protein might be expressed in this particular strain [23]. This knockout strain closely resembles the situation in homozygous mutant Rough Collies, which have a shift of the reading frame starting in exon 13. Later, a second mouse knockout strain with a deletion of exon 3 was constructed. Exon 3 is the largest coding exon and encodes the first AT-hook motif of AKNA. Both *Akna^−/−^* mouse knockout strains exhibited comparable phenotypes [23].

The genetic investigations in dogs showed a perfect genotype-phenotype correlation of the *AKNA* variant with recurrent inflammatory pulmonary disease. This association supports a causative role of *AKNA*:c.2717_2720delACAG, however cannot provide definitive proof. Nonetheless, the known role of AKNA as an important anti-inflammatory regulator of the immune response and the striking phenotypic similarities of the affected dogs with *Akna^−/−^* knockout mice in combination with the genetic association strongly suggests that *AKNA*:c.2717_2720delACAG is the causative variant.

## 5. Conclusions

We identified *AKNA*:c.2717_2720delACAG as a candidate causative variant for an inherited, autosomal recessive, recurrent inflammatory pulmonary disease in Rough Collies. The findings from knockout mice and these dogs suggest that AKNA should be considered as a candidate gene for human patients with unexplained recurrent inflammatory pulmonary disease. Our findings enabled genetic testing for Rough Collies, which can be used to avoid the unintentional breeding of affected puppies.

## Figures and Tables

**Table 1:** Results of variant filtering in an affected dog and 601 control genomes.

Filtering Step	Homozygous Variants
variants in the whole genome	3,378,300
private variants in the whole genome	1332
protein-changing private variants in the whole genome	7
protein-changing private variants in critical intervals	1

**Table 2:** Genotype-phenotype association of the *AKNA*:c.2717_2720delACAG variant.

Dogs	wt/wt	wt/del	del/del
Cases, Rough Collies (*n* = 3)	0	0	3
Controls, Rough Collies (*n* = 85)	67	18	0
Controls, various dog breeds (*n* = 539)	539	0	0

## Supplementary Materials

The following are available online at https://www.mdpi.com/2073-4425/10/8/567/s1, S1: Illumina canine_HD SNV genotypes of 10 Rough Collies, Table S1: AKNA:c.2717_2720delACAG genotypes of 539 control dogs. Table S2: Linkage and homozygosity mapping, Table S3: Accession numbers of 594 dog and 8 wolf genome sequences, Table S4: Private protein changing variants in the sequenced Rough Collie with recurrent inflammatory pulmonary disease.

## References

[1] Knowles M., Zariwala M., Leigh M. (2016). Primary ciliary dyskinesia. Clin. Chest Med..

[2] Horani A., Ferkol T.W. (2018). Advances in the genetics of primary ciliary dyskinesia: Clinical implications. Chest.

[3] Bonnefoy S., Watson C.M., Kernohan K.D., Lemos M., Hutchinson S., Poulter J.A. (2018). Biallelic mutations in LRRC56, encoding a protein associated with intraflagellar transport, cause mucociliary clearance and laterality defects. Am. J. Hum. Genet..

[4] Fassad M.R., Shoemark A., Legendre M., Hirst R.A., Koll F., le Borgne P. (2018). Mutations in outer dynein arm heavy chain DNAH9 cause motile cilia defects and situs inversus. Am. J. Hum. Genet..

[5] Merveille A.C., Davis E.E., Becker-Heck A., Legendre M., Amirav I., Bataille G., Belmont J., Beydon N., Billen F., Clément A. (2011). CCDC39 is required for assembly of inner dynein arms and the dynein regulatory complex and for normal ciliary motility in humans and dogs. Nat. Genet..

[6] Anderegg L., Im Hof Gut M., Hetzel U., Howerth E.W., Leuthard F., Jagannathan V., Leeb T. (2019). NME5 frameshift variant in Alaskan Malamutes with primary ciliary dyskinesia. PLoS Genet..

[7] Andjelkovic M., Minic P., Vreca M., Stojiljkovic M., Skakic A., Sovtic A., Rodic M., Skodric-Trifunovic V., Maric N., Visekruna J. (2018). Genomic profiling supports the diagnosis of primary ciliary dyskinesia and reveals novel candidate genes and genetic variants. PLoS ONE.

[8] Bell E.T., Griffin P., Martinello P., Robinson P. (2016). Primary ciliary dyskinesia in two English Cocker Spaniels. Aust. Vet. J..

[9] Miranda I.C., Granick J.L., Armién A.G. (2017). Histologic and ultrastructural findings in dogs with chronic respiratory disease suspected of ciliary dyskinesia. Vet. Pathol..

[10] Purcell S., Neale B., Todd-Brown K., Thomas L., Ferreira M.A., Bender D. (2007). PLINK: A tool set for whole-genome association and population-based linkage analyses. Am. J. Hum. Genet..

[11] Abecasis G.R., Cherny S.S., Cookson W.O., Cardon L.R. (2002). Merlin--rapid analysis of dense genetic maps using sparse gene flow trees. Nat. Genet..

[12] Bauer A., Jagannathan V., Högler S., Richter B., McEwan N.A., Thomas A., Cadieu E., André C., Hytönen M.K., Lohi H. (2018). *MKLN1* splicing defect in dogs with lethal acrodermatitis. PLoS Genet..

[13] Van der Auwera G.A., Carneiro M.O., Hartl C., Poplin R., Del Angel G., Levy-Moonshine A., Jordan T., Shakir K., Roazen D., Thibault J. (2013). From FastQ data to high confidence variant calls: The Genome Analysis Toolkit best practices pipeline. Curr. Protoc. Bioinformatics.

[14] Cingolani P., Platts A., Wang le L., Coon M., Nguyen T., Wang L., Land S.J., Lu X., Ruden D.M. (2012). A program for annotating and predicting the effects of single nucleotide polymorphisms, SnpEff: SNPs in the genome of *Drosophila melanogaster* strain w1118; iso-2; iso-3. Fly.

[15] Bai B., Zhao W.M., Tang B.X., Wang Y.Q., Wang L., Zhang Z., Yang H.C., Liu Y.H., Zhu J.W., Irwin D.M. (2015). DoGSD: The dog and wolf genome SNP database. Nucleic Acids Res..

[16] Jagannathan V., Drögemüller C., Leeb T. (2019). Dog Biomedical Variant Database Consortium (DBVDC). A comprehensive biomedical variant catalogue based on whole genome sequences of 582 dogs and 8 wolves. Anim Genet..

[17] Siddiqa A., Sims-Mourtada J.C., Guzman-Rojas L. (2001). Regulation of CD40 and CD40 ligand by the AT-hook transcription factor AKNA. Nature.

[18] Mao L., Yang P., Hou S., Li F., Kijlstra A. (2011). Label-free proteomics reveals decreased expression of CD18 and AKNA in peripheral CD4+ T cells from patients with Vogt-Koyanagi-Harada Syndrome. PLoS ONE.

[19] Martínez-Nava G.A., Fernández-Torres J., Martínez-Flores K., Zamudio-Cuevas Y., Clavijo-Cornejo D., Espinosa-Morales R., Lozada C.A., Gutierrez M., Granados J., Pineda C. (2018). The association of AKNA gene polymorphisms with knee osteoarthritis suggests the relevance of this immune response regulator in the disease genetic susceptibility. Mol. Biol. Rep..

[20] Manzo-Merino J., Lagunas-Martínez A., Contreras-Ochoa C.O., Lizano M., Castro-Muñoz L.J., Calderón-Corona C., Torres-Poveda K., Román-Gonzalez A., Hernández-Pando R., Bahena-Román M. (2018). The human papillomavirus (HPV) E6 oncoprotein regulates CD40 expression via the AT-hook transcription factor AKNA. Cancers.

[21] Perales G., Burguete-García A.I., Dimas J., Bahena-Román M., Bermúdez-Morales V.H., Moreno J., Madrid-Marina V. (2010). A polymorphism in the AT-hook motif of the transcriptional regulator AKNA is a risk factor for cervical cancer. Biomarkers.

[22] Lek M., Karczewski K.J., Minikel E.V., Samocha K.E., Banks E., Fennell T., O’Donnell-Luria A.H., Ware J.S., Hill A.J., Cummings B.B. (2016). Analysis of protein-coding genetic variation in 60,706 humans. Nature.

[23] Ma W., Ortiz-Quintero B., Rangel R., McKeller M.R., Herrera-Rodriguez S., Castillo E.F., Schluns K., Hall M., Zhang H., Suh W.K. (2011). Coordinate activation of inflammatory gene networks, alveolar destruction and neonatal death in AKNA deficient mice. Cell Res..

